# Learning Multirobot Hose Transportation and Deployment by Distributed Round-Robin Q-Learning

**DOI:** 10.1371/journal.pone.0127129

**Published:** 2015-07-09

**Authors:** Borja Fernandez-Gauna, Ismael Etxeberria-Agiriano, Manuel Graña

**Affiliations:** 1 Polytechnical Institute and Computational Intelligence Group, University of the Basque Country (UPV/EHU), Vitoria-Gasteiz, Spain; 2 Polytechnical Institute, University of the Basque Country (UPV/EHU), Vitoria-Gasteiz, Spain; 3 Computational Intelligence Group, University of the Basque Country (UPV/EHU), San Sebastian, Spain, & ENGINE centre Wroclaw University of Technology (WrUT), Wroclaw, Poland; Politehnica University of Bucharest, ROMANIA

## Abstract

Multi-Agent Reinforcement Learning (MARL) algorithms face two main difficulties: the curse of dimensionality, and environment non-stationarity due to the independent learning processes carried out by the agents concurrently. In this paper we formalize and prove the convergence of a Distributed Round Robin Q-learning (D-RR-QL) algorithm for cooperative systems. The computational complexity of this algorithm increases linearly with the number of agents. Moreover, it eliminates environment non sta tionarity by carrying a round-robin scheduling of the action selection and execution. That this learning scheme allows the implementation of Modular State-Action Vetoes (MSAV) in cooperative multi-agent systems, which speeds up learning convergence in over-constrained systems by vetoing state-action pairs which lead to undesired termination states (UTS) in the relevant state-action subspace. Each agent’s local state-action value function learning is an independent process, including the MSAV policies. Coordination of locally optimal policies to obtain the global optimal joint policy is achieved by a greedy selection procedure using message passing. We show that D-RR-QL improves over state-of-the-art approaches, such as Distributed Q-Learning, Team Q-Learning and Coordinated Reinforcement Learning in a paradigmatic Linked Multi-Component Robotic System (L-MCRS) control problem: the hose transportation task. L-MCRS are over-constrained systems with many UTS induced by the interaction of the passive linking element and the active mobile robots.

## Introduction

**The transportation problem** The transportation of a hose by a team of robots is a paradigmatic instance of the tasks that can be performed with *Multi-Component Robotic Systems* (MCRS) [1]. In fact, a hose with a team of robots attached to it is a Linked MCRS (L-MCRS), because it can be viewed as a collection of autonomous robots physically connected by a passive two-dimensional object, i.e. the hose.

**Autonomous learning** Reinforcement Learning (RL) [2, 3] is the main paradigm for autonomous learning. Agent-environment interaction is modeled as a Markov Decision Process (MDP) specified by a system state space, the transitions between states, and the actions that the agent can perform in each system state. Agent learning is guided by an external *reward* signal that gives a feedback assessment of the value of an action executed while the system is at some state. The goal of RL is to estimate optimal action selection policies maximizing the expected reward. In most cases, the agent-environment MDP is not an irreducible ergodic Markov Chain, but has some terminal states where the system lies forever if reached, i.e. probability of exiting the state is zero regardless of the action taken. While learning, the system must be re-initialized after reaching the terminal state in order to continue learning and exploration. Therefore, the overall learning process carried by RL is composed of successive trial episodes, which are independent realizations of the evolving MDP starting from (random) initial states, i.e. the MDP changes some of its policy making parameters in between or during trials. The simplest model-free RL algorithm is Q-learning [4, 5], which applies an iterative reward propagation rule to estimate the state-action value function implementing the optimal policy. It is often the case that rewards only happen in succesful termination states, so that learning involves the repetition of the task trial many times. Autonomous learning of the L-MCRS control by single-agent RL approaches has been demostrated [6, 7] in the small scale cases, however applying RL to systems with many robots faces an exponential computational complexity growth. Therefore, we are looking for distributed multi-agent approaches which promise computational speed-up through parallel processing, direct modeling of multi-component systems, fault-tolerance inherent to distributed control realizations, and the ability to provide linear complexity approximations to problems which are combinatorial in nature, so that their complexity grows exponentially with the number of agents.

**Overconstrained systems** In robotic applications, normal termination is given by the accomplishment of the assigned task, so that the agent obtains a positive reward. Undesired terminal states (UTS) correspond to irreversible situations where the system is stuck in a rewardless state and no evolution to a successful termination of the proposed task is possible. Hence, the work performed to reach this state is fruitless, the agent does not extract any positive training information. UTS often correspond to catastrophic error conditions. We say that a system is over-constrained when the number of UTS is large relative to the state space size. In over-constrained systems, RL convergence is severely handicapped by the high frequency of learning episodes ending in UTS. In single-agent domains, Modular State-Action Vetoes (MSAV) [8] provide a convergence speedup by early avoidance of UTS, minimizing unnecessary computation for the estimation of state-action values. The reward signal is decomposed into separate signal classes, each of them associated with a particular class of physical constraint which is commonly related to a specific subset of state variables. Modules specialized in each signal class can be taught independently when there is no interaction between state variables modeling the failure signal classes.

**MARL** Multi-Agent Reinforcement Learning (MARL) [9] scenarios involve several agents learning concurrently, so that some coordination mechanism is required for agents to agree on the joint-action to be taken. MARL has been successfully applied to several problems, such as traffic control [10], supply chain ordering management [11], prey chase [12], or intrusion detection [13]. The concurrent adaptation of the individual agent policies makes the environment non-stationary from an agent’s perspective. For this reason, few MARL algorithms offer any theoretical results about convergence to an optimal joint-policy. Moreover, solving the coordination problem ensuring convergence has additional cost in memory or communication resources [14]. The main issue in Q-Learning based MARL approaches is the exponential growth of the state-action space when the number of agents is increased (*curse of dimensionality*), because it needs to consider all possible combinations of local agent actions, even when all agents share a common set of state variables. This problem has been dealt with using general Function Approximators which reduce the amount of information storage needed [15–21], but data fitting processes impose specific assumptions which can bias the solution [17, 22]. Besides, most MARL approaches need communication between agents either for coordination or for internal computations. Communication channels introduce delays and noise compromising the system’s learning convergence. Depending on the nature of the rewards, MARL systems can be cooperative or competitive [9]. In cooperative systems, all agents receive the same reward, perceived as the result of the joint actions of the agents in the actual system state without requiring explicit communication between agents. We will only consider cooperative systems.

**Paper contribution** This paper formalizes and proves the convergence of the *Distributed Round-Robin Q-Learning* (D-RR-QL) algorithm in which agents follow a predefined Round Robin (RR) order to execute their local actions sequentially. Agents are endowed with local Q-tables performing local estimation of the state-action value function. Local agent policies are composed into a joint policy that approximates the global optimal policy using a message-based procedure. D-RR-QL converges to an optimal policy even in stochastic environments. Besides, sequential execution of local actions allow agents to use MSAV when dealing with over-constrained systems, because the outcome depends only on the local action. Computational experiments show that in over-constrained environments, D-RR-QL with MSAV is able to estimate a good approximation to an optimal policy, whereas competing state-of-the-art algorithms D-QL [23], Team QL [24], and Coordinated RL [25] never reached the goal state, even when allowed a bigger learning time span, so that they were unable to perform any learning step.

## Problem Statement

**Fig 1:**
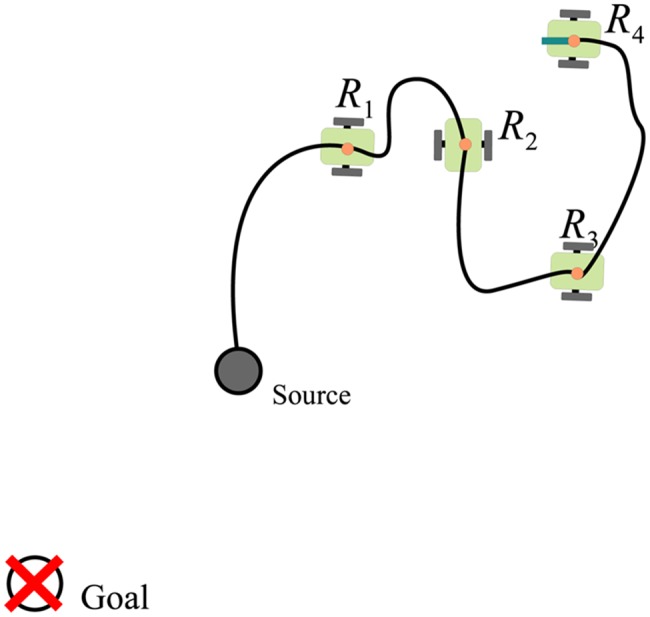
Graphic representation of the hose transportation problem. The robots attached to the hose must move coordinately so that the tip of the hose carried by the robot *R*_4_ reaches the goal position.

**Fig 2:**
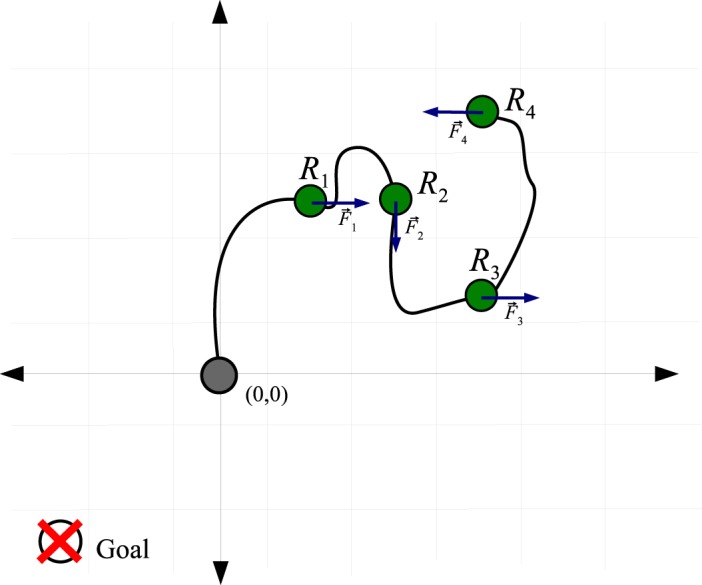
Second order model approach to the hose transportation problem applying GEDS. Arrows indicate the actual force applied by the robots on the hose.

The specific instance of the hose transportation problem dealt with in this paper is represented in Fig 1. A set of robots is attached to a hose and they must maneuver so that the tip of the hose reaches a predefined goal destination. The hose is a passive linking element that constrains non-linearly the motion of the robots. The dynamics of the whole system are highly non-linear. Accurate dynamical modeling of the hose motion pushed by the robots can be achieved using bi-dimensional Geometrically Exact Dynamic Splines (GEDS) [26–28]. The spline is defined as $\text{q}(u,t)=\sum_{nc}^{i=0}N_{i,d}(u)\cdot {\text{c}}_{i}(t)$, where **c**_*i*_(*t*), *i* = 1, …, *nc* are the dynamic control points and *N*_*i*, *d*_ is the *d*-degree polynomial specified by control point *c*_*i*_(*t*). This equation defines the position of the spline for a normalized value *u* ∈ [0,1) at time *t*. Robots are modeled as control points along the spline, and the actions executed by robots are modeled as the external forces ${\vec{F}}_{i},\;i=1,\ldots ,n$ exerted on the hose. The GEDS model is illustrated in Fig 2.

**Fig 3:**
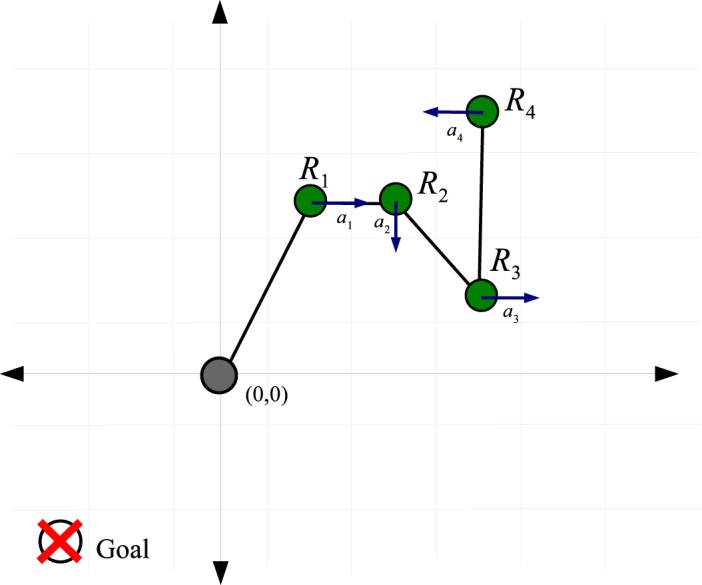
First order model approach to the hose transportation problem using line segments to represent the hose. Arrows indicate the actual force applied by the robots on the hose.

Simulation of the linked system using GEDS modeling is computationally very expensive (for some systems it takes about 2 minutes to simulate a single time step on a standard desktop computer). Transfer learning can be applied to overcome this computational cost barrier, i.e. tackling the problem as a two-step process: first, agents are trained on a simplified model that uses line segments to model the passive linking element, such as in Fig 3, so that they acquire the basic control skills through RL; second, this knowledge is transferred to learning the control of the robots when the hose is modeled by a GEDS, so that agents refine their control skills with the most accurate model [29].

Most important, the hose-robots system is an overcontrained system. Whenever any of the dynamic constraints is broken the system reaches a UTS, and it must be reset so that the learning episode needs to start again from the initial setting. Specifically, we consider four dynamic constraints: (a) the robots are not allowed to step over the hose, (b) the hose cannot be overstretched, (c) robots must not collide with each other, and (d) they must stay within the simulation bounds.

## Reinforcement Learning Background

### Single-Agent Reinforcement Learning

#### Markov decision processes

In RL algorithms, the interaction between an agent and its environment is modeled as a Markov Decision Process (MDPs), defined as a tuple < *S*, *A*, *P*, *R* >, where *S* is a finite set of states, *A* is a set of actions which the agent can execute, *P*:*S* × *A* × *S* → [0, 1] is a probabilistic transition function *P*(*s*, *a*, *s*′) giving the probability of observing state *s*′ after executing action *a* in state *s*, and *R*:*S* → ℝ is the expected immediate reward after reaching state *s*. The set of terminal states 𝓣 is defined as the set of states having null outgoing transition probabilities, i.e. 𝓣 = {*s*_*S*_∣∀*s*′ ≠ *s*, *a*;*P*(*s*, *a*, *s*′) = 0}. The action selection policy is usually modeled as the probability distribution *π*:*S* × *A* → [0, 1] of taking action *a* in state *s*.

The *value* *V* ^*π*^(*s*) of state *s* is the expected accumulated reward obtained from that state following policy *π*. It can be expressed as

$$\begin{array}{ccc} V^{\pi }(s) & = & E^{\pi }\{\underset{k=0}{\sum^{\infty }}\gamma^{k}r_{t+k+1}|s_{t}=s\}\\ \; & = & \sum_{a}\pi (s,a)\sum_{s^{\prime }}P(s,a,s^{\prime })[R(s^{\prime })+\gamma V^{\pi }(s^{\prime })], \end{array}$$ (1)

where *E* ^*π*^ denotes the expected return from time step *t* following policy *π* thereafter, *r*_*i*_ represents the reward received at time *i*, and *γ* ∈ [0, 1] is a discount-rate parameter decreasing the weight of the reward at each time-step. Eq (1) is the *Bellman equation for* *V* ^*π*^ [3]. There always exists one or more optimal policies π* maximizing the state value, denoting V* this maximum value. It satisfies:

$$\begin{array}{c} V^{*}(s)=\max_{a\in A}\{\sum_{s^{\prime }}P(s,a,s^{\prime })[R(s^{\prime })+\gamma \cdot V^{*}(s^{\prime })]\}. \end{array}$$ (2)

The goal of the learning agent is to find this optimal policy. The expected value of executing an action *a* in state *s* following a policy *π*, is modeled by the state-action value function *Q* ^*π*^(*s*, *a*):

$$\begin{array}{c} Q^{\pi }(s,a)=E^{\pi }\{\underset{k=0}{\sum^{\infty }}\gamma^{k}r_{t+k+1}|s_{t}=s,a_{t}=a\}, \end{array}$$

where *a*_*t*_ represents the action taken at time-step *t*. The optimal state-action value function is obtained applying an optimal policy π*:

$$\begin{array}{c} Q^{*}(s,a)=\sum_{s^{\prime }}P(s,a,s^{\prime })\{R(s,a,s^{\prime })+\gamma [\max_{a^{\prime }}Q^{*}(s^{\prime },a^{\prime })]\}. \end{array}$$ (3)

An episode consists of a sequence of state transitions fired by actions executed by the agent, leading to a terminal state that represents the success/failure of the completion of a proposed task. The deterministic *greedy* action selection involves selecting always the action with the highest Q-value *exploiting* the available knowledge

$$\begin{array}{c} \pi_{greedy}(s)=\underset{a^{\prime }\in A}{\arg\;\max}Q(s,a^{\prime }), \end{array}$$

implementing the optimal policy implied by the actual Q-table. This policy is applied in the test and operational phases of the system. In order to ensure convergence during learning, the system must be able to explore, i.e. to reach any state from any state, therefore action selection probabilities must be non-null. One way to ensure that is an stochastic action selection algorithm such as *Soft-Max*:

$$\begin{array}{c} \pi_{SoftMax}(s,a,\tau )=\frac{\exp^{Q(s,a)/\tau }}{\sum_{a^{\prime }}\exp^{Q(s,a^{\prime })/\tau }}, \end{array}$$ (4)

where *τ* ∈ (0,1] is a parameter controlling the flatness of the probability distribution, aka temperature parameter.

#### Q-Learning

Q-Learning [5] is a model-free Temporal Difference reinforcement learning algorithm performing an iterative estimation of the state-action value function *Q*(*s*, *a*), hence its name. It allows agents to find the optimal policy for an MDP without *a priori* knowledge about the transition function *P* and the reward function *R*. Denote {*Q*_*t*_(*s*, *a*);*t* = 0, 1, 2, …} the sequence of state-action value function estimations during RL, converging to Q*(*s*, *a*).

The original Q-Learning algorithm stores in tabular form state-action values *Q*(*s*, *a*). At each learning episode, the agent observes the actual state *s*_*t*_, selects an action to be executed *a*_*t*_, receives the immediate reward *r*_*t*_, observes the subsequent state *s*′ and updates the state-action value matrix to *Q*_*t*_(*s*, *a*) from the previous estimation *Q*_*t*−1_(*s*, *a*). Learning speed is controlled by the gain *α*_*t*_ and Q-values are updated using the following rule:

$$\begin{array}{c} Q_{t}\left(s,a\right)\leftarrow (1-\alpha_{t})Q_{t-1}\left(s,a\right)+\alpha_{t}[r_{t}+\gamma \max_{a^{\prime }}Q_{t-1}(s^{\prime },a’)] \end{array}$$ (5)

Q-learning converges with probability 1 to the optimal policy in a stationary environment [5] when all system’s states are visited infinitely often, and the learning gain *α*_*n*_ complies with the stochastic gradient conditions. This ergodic condition on the stochastic process realized by the agent according can not be fulfilled by an MDP containing terminal states (𝓣 ≠ ⌀) or having sparse state transition probability matrix. To avoid the latter, SoftMax action selection function is applied. To overcome the former, the learning process must be reinitialized each time that it falls in a terminal state. A separate representation for each state-action value is also required, and thus, the storage space requirements are *O*(∣*S* × *A*∣), growing linearly with the state-action product space size.

### Multi-agent Reinforcement Learning

MARL algorithms were classified in [30] depending on agents’ perception capabilities: in *Independent Learners* (IL) approaches, agents are able to observe the global state but restricted to know their own local actions, while in *Joint-Action Learners* (JAL) approaches, agents are allowed to observe both the global state and all the actions taken by all agents. IL are a more realistic framework for real life deployment of MCRS systems, because communication requirements are inmediately scalable.

#### Independent Learners (IL)

IL approaches decompose the global state-action value Q-table into local Q-tables owned by agents. The easliest instance of this approach is the naive IL approach [30], where agents implement a single-agent RL algorithm obviating the decisions and the payoff obtained by the remaining agents. Because of the lack of a coordination mechanism, the system cannot be guaranteed to converge to a stable or a globally optimal policy. The *Distributed Rewards* and *Distributed Value Functions* were studied in [31] as a way to motivate cooperation between neighbors. Instead of updating the state-action values using only the local reward or values, agents also used weighted rewards or state-action values of their teammates. The computational complexity of these methods scale linearly with the number of agents, but no proof of convergence exists. *Distributed Q-Learning* (D-QL) [23] performs local training on a local Q-table *per* agent assuming optimal behavior from all remaining agents. The virtual global Q-table is decomposed into local Q-tables. For each local action *a*_*i*_, the agent stores only the value of the joint-action containing *a*_*i*_ that maximizes the reward. Thus, agents need not be aware of the actions taken by the rest of agents to be able to converge to a globally optimal policy. However, this convergence is not guaranteed in stochastic environments. Some authors propose the use of an actual centralized table updated by all agents [32, 33]. Some others have presented very good results applying a heuristically modified version of D-QL, known as Hysteretic QL [34]. Distributed RL has also been proposed to deal with multi-objective optimization problems using negotiation protocols between agents [35].

#### Joint-Action Learners (JAL)

The most straight-forward JAL approach to MARL is to implement an independent Q-Learning process in each agent estimating the Q-value of each of the joint-actions. *Team Q-Learning* [24] (Team-QL) assumes that a unique optimal action decision exists in each state.

Some authors have proposed model based heuristic algorithms [30, 36] to estimate the most likely response of the remaining agents, using them to bias local policies towards coordinated joint actions. Those models are learned from experience. Following a different approach, each state in an MDP can be regarded as a virtual stateless Stochastic Game (SG), therefore adaptive methods [14, 37] have been proposed to bias local action selection towards a globally optimal joint action. These approaches require additional memory resources and knowledge about the optimal state-joint-action function, scaling badly with the problem size.

The *Coordinated Reinforcement Learning* (Coordinated-RL) [25] approximates the global joint value function as a linear combination of local value functions [38]. The complexity of agreeing on a globally optimal joint action can be reduced assuming that agents need not coordinate with all remaining agents, but with a small subset. These coordination dependencies between agents are context-specific, can change dynamically and can be defined as a *Coordination-Graph*, where undirected edges represent a coordination dependence between agents. The use of the Coordination Graph reduces the state-action space by defining which actions are relevant to each local value function, and it can still be further reduced by identifying which state variables are relevant to each local value function.

## Related Work

In this section, we first review previous work on dynamic constraints in single-agent RL. To the best of our knowledge, no relevant work can be found on constraints in multi-agent systems. Next, we describe the three MARL algorithms that we have used in our experiments for benchmarking: Team Q-Learning, Distributed Q-Learning, and Coordinated RL.

### Dynamic Constraints in RL

In the literature about RL, undesired terminal states (UTS) have been dealt with in different ways: (a) Using null state transitions and/or generating negative rewards [39–41]. (b) Manually discarding actions that could lead to an undesirable state [42], defining a state dependent action repertoire *A*(*s*). (c) Defining MDPs with Constraints [43–45], where the goal is to maximize the expected accumulated reward subject to minimize the risk of ending in an *error state* after taking action *a* in state *s*. The estimated risk expectation is updated similarly to the Q-table values. The state-action value is the weighted mixture of expected accumulated rewards and risks. Learning searches first for a minimum risk policy. After that, an optimal policy below an upper bound for the allowed risk of reaching an error state is sought. (d) Assuming availability of experience tuples from a demonstration of the task performed by an expert [46]. Using state-actions known to be safe from these initial samples, RL is used to achieve a near-optimal performance with respect to the available data. Finally, (e) [47] consider undesirable states as critical, proposing a graph-based algorithm to safely explore a given state-space without reaching a *critical state*. They assume that error states are defined by the magnitude of the rewards, which involves that states *near* critical states can be detected by the rewards. None of these approaches are feasible for over-constrained systems such as L-MCRS for the following reasons: (a) using null state transitions and negative rewards does not encorage the agent to avoid them fast enough because Q-values are weighted sums of the immediate negative rewards and future positive rewards, (b) the system designer requires knowledge of the transition function in order to manually define a state dependent action repertoire, and (c) calculating the risk of reaching an UTS from each state-action pair makes the problem even more complex and requires even more computation.

#### Modular state-action vetoes

In over-constrained single-agent tasks, we define a partition of the terminal states into two sets 𝓣 = *G*∪*U*, where *G* ⊆ *S* is the set of good termination conditions, and *U* ⊆ *S* is the set of UTS. We can also identify the set *T* ⊆ *S* of transitory states. We can state that they are characterized by the sign of the achieved rewards as follows:

$$\begin{array}{ccc} G & = & \{s|s\in S,R(s)>0\},\\ T & = & \{s|s\in S,R(s)=0\},\\ U & = & \{s|s\in U,R(s)<0\}. \end{array}$$

We can decompose further the reward signal as *m* different signals

$$\begin{array}{c} R(s)=R^{G}+\underset{i=1}{\sum^{m-1}}R_{i}^{U}(s), \end{array}$$

where

- *R* ^*G*^(*s*) ≥ 0 is strictly positive only if the task has been successfully accomplished, and zero for transitory states.
- $R_{i}^{U}\left(s\right)\leq 0,i=1,\ldots ,m-1$ are strictly negative signals only when a certain class of UTS has been reached, e.g. collision, broken physical constraint, etc.

This modular partition into is particularly useful when dealing with over-constrained systems, because it facilitates the learning of how to avoid triggering of the alarm signals $R_{i}^{U}$ using only information from the relevant state subspace $S_{i}^{U}$. Under the assumption that the optimal policy will in no case reach a UTS, Safe Modular State-Action Veto (MSAV) policies [8] are useful to boost the exploration efficiency. Let the state space be $S=S_{X}\times S_{i}^{U}$, where $S_{i}^{U}$ is the state subspace used by the *i*-th module. Every time $R_{i}^{U}$ is triggered, ∣*S*_*X*_∣ states are effectively vetoed in the complete state space. Thus, MSAV policies will learn *A* ^*e*^(*s*) faster than any policy learning over the complete state space, and the speed gain introduced is proportional to the number of irrelevant state variables.

The system learns simultaneously the Q-values and the *Safe Action Repertoire *A*^*e*^(*s*)*

$$\begin{array}{c} A^{e}(s)=\{a|a\in A\wedge (\sum_{s^{\prime }\in U}P(s,a,s^{\prime })=0)\}, \end{array}$$

which is the set of actions that cannot lead to a UTS. Vetoes are imposed to risky state-action pairs using only the corresponding state subspace. UTS can be safely avoided and learnt using a Safe-MSAV policy such as

$$\begin{array}{c} {\hat{\pi }}_{SoftMax}(s,a,\tau )=\{\begin{array}{cc} 0 & Veto(s,a)\\ \frac{\exp^{Q(s,a)/\tau }}{\sum_{a^{\prime }\in A^{e}(s)}\exp^{Q(s,a^{\prime })/\tau }} & \neg Veto(s,a) \end{array}, \end{array}$$ (6)

where *Veto*(*s*, *a*) is a boolean value that represents whether state-action pair (*s*, *a*) should be available to be chosen by the agent or vetoed based on the agent’s past experience. Using a Safe-MSAV policy can converge with probability 1 to the optimal values of MDP < *T*∪*G*, *A* ^*e*^, *P*, *R* > using standard Q-Learning.

### Cooperative Multi-Agent Reinforcement Learning

When the system is composed of several interacting autonomous agents, the Multi-Agent Reinforcement Learning (MARL) problem consists of the search for the joint optimal policies maximizing the reward for each and all agents [9]. In the following, regular characters denote local actions (i.e. *a*, *a*_*i*_, *A*) and corresponding functions (*Q*, *P* and *R*); bold characters denote joint-actions (i.e. **a**, **a**_*i*_, **A**) and corresponding functions. The extension of the MDP for the multi-agent case is a Stochastic Game (SG) defined by tuple < *S*, *A*_1_, …, *A*_*N*_, **P**, **R**_1_, …, **R**_*N*_ >, where *N* is the number of agents, *S* is the set of environment states, *A*_*i*_, *i* = 1, …, *N* are sets of actions that each agent can execute, yielding the joint action set **A** = *A*_1_ × … × *A*_*N*_. **P**:*S* × **A** × *S* → [0, 1] is the probabilistic state transition function **P**(*s*,**a**, *s*′) that defines the probability of observing state *s*′ after all agents execute the joint action **a** ∈ **A** in state *s*, and **R**:*S* × **A** × *S* → ℝ^*N*^ are the expected rewards received by agents after transition (*s*,**a**, *s*′).

In the multi-agent case, state transitions are the result of the concurrent joint action **a**_*n*_ = [*a*_1,*n*_, …, *a*_*N*, *n*_]^*T*^ ∈ **A**, *a*_*i*, *k*_ ∈ *A*_*i*_. As a consequence, the rewards also depend on the collective action. The *local policy* of each agent *π*_*i*_:*S* × *A*_*i*_ → [0, 1] gives the probability of the *i* ^*th*^ agent executing action *a* in state *s*. The local policies are composed into a global *joint policy* **π**(*s*,**a**) = {*π*_1_(*s*, *a*_1_), …, *π*_*N*_(*s*, *a*_*N*_)}.

The SG is fully *cooperative* when the local rewards are identical for all agents **R**_1_(*s*,**a**, *s*′) = **R**_2_(*s*,**a**, *s*′) = … = **R**_*N*_(*s*,**a**, *s*′),

If a centralized learner exists, the task could be mapped to an MDP, whose centralized Q-function could be expressed as

$$\begin{array}{c} Q^{\pi }(s,a)=E^{\pi }\{\underset{k=0}{\sum^{\infty }}\gamma^{k}r_{t+k+1}|s_{t}=s,a_{t}=a\}, \end{array}$$ (7)

and the optimal policy could be expressed as ${\text{π}}^{*}\left(s\right)=\left\{\pi_{1}^{*}\left(s\right),\ldots ,\pi_{N}^{*}\left(s\right)\right\}$, where

$$\begin{array}{c} \pi_{i}^{*}(s)=\arg\max_{a_{i}}Q^{*}(s,[a_{1}\ldots a_{i}\ldots a_{N}]), \end{array}$$ (8)

where *a*_*i*_ ∈ *A*_*i*_ and *i* = 1, …, *N*. When there are more than one optimal action choice, the optimal policy is achieved by a consensus protocol.

*Team Q-Learning* (*Team-QL*) [24] works on the assumption that there is only one optimal state-action value on each state, so that it updates the local estimates using the standard Q-Learning update rule Eq (5). The Q function depends on the complete joint-action **a** ∈ **A**, and thus, storage requirements grow with 𝓞(*S* × **A**).

In *Distributed Q-Learning (D-QL)* [23] each agent assumes that the remaining agents will select the optimal action. The virtual centralized state-action Q function Eq (7) is decomposed into smaller local *Q* ^*i*^(*s*, *a*) where *a* ∈ *A*_*i*_, such that only the maximum value for each local action is stored:

$$\begin{array}{c} Q^{i}(s,a)=\max_{a_{i}}Q(s,[a_{1}\ldots a_{i}\ldots a_{N}]). \end{array}$$

This algorithm needs only to be aware of the local action, updating the local Q-values only when the updated state-action value is higher than the previous one:

$$\begin{array}{c} Q_{t}^{i}(s,a)\leftarrow \max\{Q_{t-1}^{i}(s,a),r_{t}+\gamma \max_{a^{\prime }}Q_{t-1}^{i}(s^{\prime },a^{\prime })\}. \end{array}$$

Because Q-values are only updated when increased, D-QL is expected to estimate the same optimal policies that a centralized learner would learn, without any explicit communication between agents. Its storage requirements grow with 𝓞(*S* × *A*).

More sophisticated approaches include explicit coordination mechanisms. *Coordinated RL* (C-RL) [25] approximates the global joint value function as a linear combination of local value functions [38]. The complexity of agreeing on a globally optimal joint action can be reduced assuming that agents need not coordinate with all remaining agents, but with a smaller subset. These coordination dependencies are defined as a *Coordination-Graph*, denoted *CG*(*s*) = {*V*, *E*}, where undirected edges *e*_*ij*_ ∈ *E* represent a coordination dependence between agents *i* and *j*. Each agent has a local *Q*_*i*_ function, which contributes to the global function $Q=\sum_{i=1}^{N}Q_{i}\left(s_{i},a\right)$, where *a* ∈ × *A*_*j*_ such that *e*_*ij*_ exists in *CG*(*s*). This method reduces the state-action space by defining which actions are relevant to each *Q*_*i*_ function.

When implementing the optimal policy, agents need to coordinate the selection of the local actions to build the optimal joint-action. Agents can agree on the optimal joint-action using a *Variable Elimination* (VE) procedure, which maximizes the global value function by maximizing one variable at a time. An agent is chosen to communicate its expected local value for each action to one of its neighbors. Then, this agent can be eliminated from the graph and the selected neighbor can compute the action that maximizes its local value function for the one chosen by the first agent. This procedure is applied to the remaining agents. When only one agent is left, it computes the global maximum and the joint action is propagated with another pass over the CG. This algorithm can be implemented using a simple message-based protocol, and it always computes the global optimal joint-action regardless of the elimination order. However, time constraints can render this approach not suitable for real-time systems.

## Cooperative Round-Robin Stochastic Games

To reduce the complexity of dealing with joint actions, we propose to decompose them into a sequence of local actions by considering a Round-Robin schedule assigning turns to execute actions. The Cooperative Round-Robin Stochastic Games formalize this idea.

**Definition 1** A *Cooperative Round-Robin Stochastic Game* (C-RR-SG) is a tuple < *S*, *A*_1_…*A*_*N*_, *P*, *R*, *δ* >, where

- *N* is the number of agents.
- *S* is the set of states, fully observable by all the agents.
- *A*_*i*_, *i* = 1, …, *N* are the sets of local actions to the *i*-th agent.
- *P*:*S* × ∪*A*_*i*_ × *S* → [0, 1], *i* = 1, …, *N* is the state transition function *P*_*t*_(*s*, *a*, *s*′) that defines the probability of observing *s*′ after agent *δ*(*t*) executes, at time *t*, action *a* from its local action repertoire *A*_*δ*(*t*)_.
- *R*:*S* × ∪*A*_*i*_ × *S* → ℝ is the shared scalar reward signal *R*_*t*_(*s*, *a*, *s*′) received by all agents after executing a local *a* action from *A*_*δ*(*t*)_.
- *δ*:ℝ → {1, …, *N*} is the turn function implementing the Round-Robin cycle of agent calling for action execution. *δ*(*t*) gives the index of the agent allowed to execute an action at time *t* (times are relative to the start of the episode). This function is cyclic, ∀*t* ∈ ℕ;*δ*(*t*) = *δ*(*t* + *N*), therefore, agents are always visired in the same order, so that without loss of generality, we can denote *i* + 1 the agent that will be visited after *i*.

The state value function *V* ^**π**^(*s*, *i*) gives the value of being in state *s* for agent *i* ∈ {1, …, *N*} under joint policy **π**, and it is the expected accumulated discounted rewards following the joint policy **π**. The Bellman equation for a joint policy **π** in a C-RR-SG is

$$\begin{array}{ccc} V^{\pi }(s,i) & = & E^{\pi }\{\underset{k=0}{\sum^{\infty }}\gamma^{k}r_{t+k+1}|s_{t}=s\}\\ \; & = & \sum_{a\in A_{i}}\pi_{i}(s,a)\sum_{s^{\prime }}P(s,a,s^{\prime })\\ \; & \cdot & [R(s,a,s^{\prime })+\gamma V^{\pi }(s^{\prime },i+1)], \end{array}$$

where *E* ^**π**^ represents the expectation from time *t* onwards, following joint policy **π**. The optimal state value V*(*s*, *i*) is given by:

$$\begin{array}{c} V^{*}(s,i)=\max_{a\in A_{i}}\{\sum_{s^{\prime }}P(s,a,s^{\prime })[R(s,a,s^{\prime })+\gamma V^{*}(s^{\prime },i+1)]\}. \end{array}$$ (9)

The state-action value function for agent i following joint policy **π** can be expressed as

$$\begin{array}{c} Q^{\pi }(s,a,i)=\sum_{s^{\prime }}P(s,a,s^{\prime })[R(s,a,s^{\prime })+\gamma V^{\pi }(s^{\prime },i+1)] \end{array}$$ (10)

and the optimal state-action value function for each agent *i* is

$$\begin{array}{c} Q^{*}(s,a,i)=\sum_{s^{\prime }}P(s,a,s^{\prime })[R(s,a,s^{\prime })+\gamma \max_{a^{\prime }\in A_{j}}Q^{*}(s^{\prime },a^{\prime },i+1)]. \end{array}$$

## Centralized Q-Learning for C-RR-SG

The straightforward approach to learn the optimal policy for a C-RR-SG is applying the single-agent Q-Learning update:

$$\begin{array}{ccc} Q_{t}\left(s,a,\delta (t)\right) & \leftarrow & (1-\alpha_{t})Q_{t-1}\left(s,a,\delta (t-1)\right)\\ & & +\;\alpha_{t}[r_{t}+\gamma \max_{a^{\prime }}Q_{t-1}(s_{t+1},a^{\prime },\delta (t-1))]. \end{array}$$ (11)

**Proposition 1** A centralized single-agent Q-Learning training of a C-RR-SG by the rule of Eq (11) will converge to Q*(*s*, *a*, *i*) if each agent fulfills the conditions for convergence of the single agent Q-learning.

*Proof*. If we are able to map the C-RR-SG into an MDP, then the proof of the proposition follows from the convergence of single agent Q-learning. The map is as follows: The MDP state space S is the same of the C-RR-SG, since it is defined by a set of global variables visible by all agents. The MDP set of actions is the union of the local sets of actions $A={⋃}_{i=1}^{N}A_{i}$, where actions lose the direct identification with the agent. The MDP state transition probability is built by considering *P*(*s*, *a*, *s*′) = *P*(*s*, *a*_*δ*(*t*)_, *s*′), that is, actions are sequenced according to the turn function. The MDP reward function is built by *R*(*s*′) = *R*(*s*, *a*_*δ*(*t*)_, *s*′), that is, we record the reward at the arriving state. The C-RR-SG turn function removes the uncertainty and concurrency about the joint agent actions, therefore the MDP policy can be stated as *π*(*s*_*t*_) = **π**_*δ*(*t*)_(*s*_*t*_), that is, we apply at each time the local policy of the agent selected by the turn function. The MDP Q-table is built by collapsing all the agent Q-tables into a monolithic one by applying the turn function, i.e. *Q*_*t*_(*s*, *a*) = *Q*(*s*, *a*, *δ*(*t*)). Single-agent Q-Learning converges to optimal Q*(*s*, *a*) values under two conditions [5]: all state-action pairs (*s*, *a*), *a* ∈ ∪*A*_*i*_, *s* ∈ *S* may be visited infinite times, and the learning gain *α*_*t*_ is decreased according to the conditions for convergence of the stochastic gradient, i.e. ∑_*t*_ *α*_*t*_ = ∞ and ∑_*t*_(*α*_*t*_)^2^ < ∞. The second condition is easy to fulfill, by selecting an appropriate schedule for *α*_*t*_. To prove the first condition, it is enough to show that the MDP obtained from the C-RR-SG is an irreducible Markov chain. This follows inmediately if the sate transition matrix *P* does not contain zero elements, and the action selection policy attributes nonzero probability to all actions in every state. The latter is assured by the use of exploratory policies (i.e. SoftMax), the former is ensured by the fact that each individual agent fullfills the conditions for convergence, i.e. *P*(*s*, *a*_*δ*(*t*)_, *s*′) > 0 therefore the MDP state transition probabilities will be positive.

This centralized learning process represents the straightforward tabular implementation of centralized Q-Learning on a C-RR-SG. This algorithm is able to learn a set of locally optimal policies which result in a globally optimal policy π*. This centralized implementation, while conceptually simple, has a big drawback: it assumes an omniscient centralized learner. This is hardly true in real-world applications because of the communication requirements, especially in an MCRS scenario, where noisy and faulty communications make this problem even worse. From the point of view of computational complexity, this centralized learner should store ∣*S* × ∪*A*_*i*_∣, *i* = 1, …, *N* entries in its Q-table.

## Distributed Round-Robin Q-Learning

In distributed implementations the state-action value matrix *Q*(*s*, *a*, *i*) is decomposed into local independent matrices *Q* ^*i*^(*s*, *a*) corresponding to the agent owning turn *i*, so that all information is distributed *Q*(*s*, *a*, *i*) = *Q* ^*i*^(*s*, *a*). We present two different distributed Round-Robin Q-Learning algorithms (D-RR-QL) update rules for stochastic games of type C-RR-SG that differ in their communication strategies: the first one requires at each time-step to send information from the current agent to the next in turn according to the Round-Robin schedule, the second is communication-free. In both cases, we prove convergence to an optimal policy.

In the first D-RR-QL update rule, the Q-table is updated using the information from the next agent in the RR scheduling cycle, so that local Q-table updating does not need to wait the full cycle. However it requires that agent *i* + 1 informs agent *i* of its optimal policy given by $\underset{a^{\prime }}{\text{max}}Q_{t-1}^{j}\left(s_{t+1},a^{\prime }\right)$. Once this value is communicated, the *i*-th agent can update its own Q-table using the following update rule:

$$\begin{array}{ccc} Q_{t}^{i}\left(s,a\right) & \leftarrow & (1-\alpha_{t})Q_{t-1}^{i}\left(s,a\right)\\ & & +\;\alpha_{t}[r_{t}+\gamma \max_{a^{\prime }}Q_{t-1}^{j}(s_{t+1},a^{\prime })]. \end{array}$$

The D-RR-QL algorithm using this rule inherits its convergence properties from the centralized single-agent algorithm, and needs not additional proof.

The communication-free D-RR-QL algorithm is specified in Algorithm 1. In this algorithm the local *Q* ^*i*^ table is updated at the end of an RR cycle using the information of the rewards that have been broadcasted to all agents because we are dealing with a fully cooperative MARL, according to the following rule:

$$\begin{array}{ccc} Q_{t}^{i}(s,a) & = & (1-\alpha_{t})Q_{t-N}^{i}\left(s,a\right)\\ & & +\;\alpha_{t}[\underset{k=0}{\sum^{N-1}}\gamma^{k}r_{t+k}+\gamma^{N}\max_{a^{\prime }}Q_{t}^{i}(s_{t+N},a^{\prime })] \end{array}$$ (12)

applied when *s*_*t*_ = *s*, *a*_*t*_ = *a*, *δ*(*t*) = *δ*(*t* − *N*) = *i*. This update rule allows each agent to update its local *Q* ^*i*^-table without the need to know the Q-tables of other agents. This is the communication-free implementation of the D-RR-QL algorithm which will be the subject of our experiments.

**Algorithm 1** Algorithm executed by agent *i* for the estimation of the local *Q* ^*i*^ table according to the communication-free D-RR-QL algorithm assuming a global time counter visible to all agents.

Initialize $\left[Q_{0}^{i}(s,a,i)=0;s\in S;a\in A;i=1\ldots N\right]$ arbitrarily

  Repeat (for each episode) *n*:

    Repeat (for each step *t* of episode):

- *Wait until* *δ*(*t*) = *i*
- Observe current state *s*_*t*_
- Select and execute action *a*_*t*_
- *Give turn to next agent in RR*
- Observe the rewards *r*_*t*_, …, *r*_*t* + *N*−1_ of a complete RR cycle and new state *s*_*t* + *N*_ after the RR cycle.
- Adaptation of the local *Q* ^*i*^ table
  – if *s*_*t*_ = *s*, *a*_*t*_ = *a*, *i* = *δ*(*t*)
    $$\begin{array}{ll} Q_{t}^{i}(s,a) & =\left(1-\alpha_{t}\right)Q_{t-1}^{i}(s,a)\\ & +\;\alpha_{t}\left[\underset{k=0}{\overset{N-1}{\sum }}\gamma^{k}r_{t+k}+\gamma^{N}\underset{a^{\prime }}{\max}Q_{t}^{i}(s_{t+N},a^{\prime })\right] \end{array}$$
  – $Q_{t}^{i}(s,a)=Q_{t-1}^{i}(s,a)$ otherwise

  until *s*_*t* + 1_ is terminal

**Proposition 2** *Convergence of the communication-free D-RR-QL of Algorithm 1 to the optimal policy*, $Q_{t}^{i}\left(s,a\right)\to Q^{*}\left(s,a,i\right)$ *as *t* → ∞, for a given a C-RR-SG ⟨*S*, *A*_1_…*A*_*N*_, *P*, *R*, *δ*⟩ is guaranteed when each agent fulfills the conditions of convergence of single-agent Q-Learning in a MDP*.

*Proof*. Let us consider for agent *i* the state value after N steps, combining the accumulation of the sequence of *N* rewards observed during an RR cycle starting and ending in agent *i* and the forgetting factor, which is given by

$$\begin{array}{c} R_{t}^{\left(N\right)}=\underset{k=0}{\sum^{N-1}}\gamma^{k}r_{t+k+1}+\gamma^{N}V_{t}(s_{t+N},i). \end{array}$$

For agent *i*, the *corrected N-step truncated reward return* [3], which is is the perceived increment of the state value after N update steps of the learning process is given by:

$$\begin{array}{c} \Delta V_{t}(s_{t},i)=\alpha [R_{t}^{\left(N\right)}-V_{t}(s_{t},i)], \end{array}$$ (13)

where *V*_*t*_(*s*_*t*_, *δ*(*t*)) is the value of being in state *s*_*t*_ at time *t*. For single agent learning, N-step TD Methods converge to optimal value functions V*(*s*) due to their *error reduction property* [4]:

$$\begin{array}{c} \max_{s}|E^{\pi }\{R_{t}^{\left(N\right)}|s_{t}=s\}-V^{*}(s)|\leq \gamma^{N}\max_{s}|V_{t}(s_{t})-V^{*}(s)|, \end{array}$$

which means that the error commited by worst one-step ahead estimate *V*_*t*_(*s*_*t*_) is an upper bound of the error committed by the N-step ahead estimate $E^{\text{π}}\left\{R_{t}^{\left(N\right)}|s_{t}=s\right\}$ of the optimal state value function. Besides, this error bound can be made as tight as desired because the factor *γ* ^*N*^ → 0 as *N* → ∞. From Eq (13) we can derive the update rule for the state-action table of Eq (12) used by Algorithm 1:

$$\begin{array}{ccc} \Delta Q_{t}^{i}(s_{t},a) & = & \alpha [\underset{k=0}{\sum^{N-1}}\gamma^{k}r_{t+k+1}+\gamma^{N}V_{t}(s_{t+N},i)]\\ & = & \alpha [\underset{k=0}{\sum^{N-1}}\gamma^{k}r_{t+k+1}+\gamma^{N}\max_{a^{\prime }}Q_{t}^{i}(s_{t+N},a^{\prime })]. \end{array}$$

Therefore, in the communication-free D-RR-QL of Algorithm 1 each agent performs a N-step ahead update of its local state-action value function, where the accumulated rewards are exogenous variables to the agent. The local updating of *Q* ^*i*^(*s*, *a*) are time-interleaved Q-learning processes, which converge to the optimal Q*(*s*, *a*, *i*) if the stochastic gradient conditions of [5] hold for each agent, i.e. it can visit all state-action pairs infinitely often. Each agent computations are separated from the others, though they share the same global state and rewards so they can not be considered statistically independent. Statistical independence is not a requirement for convergence of the computationally independent learning processes.

## Composition of Concurrent Joint-Action Policies

A C-RR-SG is an approximation to the original Cooperative Stochastic Game (C-SG) problem, because agents are forced to carry out actions following a predefined sequential order. Policies learned using this approximation are very likely to be suboptimal in the original C-SG environment, because the discounted reward approach assumes that the task completion instant is critical for the definition of optimal policies. The problem posed in this section is how to compose the local policies learned by the separate agents using D-RR-QL into the optimal policy for the original C-SG, where actions can be executed concurrently. Therefore, we deal with a combinatorial optimization problem. In this section, we present a sequential greedy coordination algorithm to build an approximation of the C-SG optimal policy **π**(*s*,**a**) from the distributed Q-tables learned using D-RR-QL.

**Definition 2** A *C-RR-SG* < *S*, *A*_1_…*A*_*N*_, *P*, *R*, *δ* > is a *sequential realization* of a *C-SG* < *S*, *A*_1_…*A*_*N*_,**P**,**R** > if the following two properties hold:

- ∀*s*, *s*′,**a**, *i*;**P**(*s*,**a**_*i*_, *s*′) = *P*(*s*, *a*_*i*_, *s*′)
- ∀*s*, *s*′,**a**, *i*;**R**(*s*,**a**_*i*_, *s*′) = *R*(*s*, *a*_*i*_, *s*′),
  where joint-action **a**_*i*_ is defined as the joint-action in which only the *i*-th agent performs an action, i.e. **a**_*i*_ = [∅, …, ∅, *a*_*i*_, ∅, …, ∅]. Null action ∅ is defined as the action that does not perform any state transition: ∀*s*;*P*(*s*, ∅, *s*) = 1.

The transition probabilities and *discounted* rewards of a joint-action **a** = [*a*_1_, *a*_2_, …, *a*_*N*_], *a*_*i*_ ∈ *A*_*i*_, are approximated by the sequential execution of agents’ actions following RR scheduling *δ*:

- **P**(*s*,**a**, *s*′) ≃ *P*(*s*, *a*_*δ*(1)_, *a*_*δ*(2)_…*a*_*δ*(*N*)_, *s*′)
- **R**(*s*,**a**, *s*′) ≃ *R*(*s*, *a*_*δ*(1)_, *a*_*δ*(2)_…*a*_*δ*(*N*)_, *s*′),

where *P*(*s*, *a*_*δ*(1)_, *a*_*δ*(2)_…*a*_*δ*(*N*)_, *s*′) and *R*(*s*, *a*_*δ*(1)_, *a*_*δ*(2)_…*a*_*δ*(*N*)_, *s*′) denote the probability of reaching state *s*′ after executing actions specified in **a** in the sequence established by *δ*, and the expected reward of this transition, respectively. Under this assumption, Q-values for joint-actions can be approximated by composition of the transition and reward functions of local actions. The state-action value for composed joint-actions can be defined as

$$\begin{array}{ccc} Q^{\pi }(s_{0},a) & = & \sum_{s_{1}\ldots s_{N}\in S}(\underset{i=0}{\prod^{N-1}}P(s_{i},a_{i+1},s_{i+1}))\\ & \cdot & \{\underset{i=0}{\sum^{N-1}}\gamma^{i}\cdot R(s_{i},a_{i+1},s_{i+1})\\ & + & \gamma^{N}\cdot \max_{a^{\prime }}Q^{\pi }(s_{N},a^{\prime },\delta (1))\} \end{array}$$ (14)

where **a** = [*a*_1_ *a*_2_…*a*_*N*_], *a*_*i*_ ∈ *A*_*δ*(*i*)_. Note that rewards inside a joint-action are weighted as in the C-RR-SG.

**Greedy selection of optimal joint policy** The combinatorial optimization problem of chosing the best ordering of the local actions has been tackled as a greedy variable selection process [25] requiring agents to store estimations of the transition probabilities ${\hat{P}}_{i}\left(s,a,s^{\prime }\right)$ and rewards ${\hat{R}}_{i}\left(s\right)$ for local actions *a* ∈ *A*_*i*_. Observed rewards can be stored with minimal storage requirements. Typical delayed reward scenarios involve a small number of terminal states that receive a non-null reward. Moreover, MSAV policies store state-action pairs with negative rewards using only the relevant state variables. Therefore, an agent can keep track of those states that have yielded an immediate positive reward in the past as a set of pairs $S_{i}^{r}=\left\{\left\langle s_{i},r_{i}\right\rangle ;i=1,\ldots ,k\right\}$, where *k* is the number of elements in the set. Every time a reward *R*(*s*, *a*, *s*′) > 0 is observed, the pair ⟨*s*′, *R*(*s*, *a*, *s*′)⟩ is added to the set $S_{i}^{r}$, if it wasn’t already in it. The reward function estimate can be written as

$$\begin{array}{c} {\hat{R}}_{i}(s)=\{\begin{array}{cc} r_{i} & if\;\exists i;1\leq i\leq k\wedge s_{i}=s\\ 0 & otherwise, \end{array}, \end{array}$$

where *s*_*i*_ represents the *i* ^*th*^ state in set *S*_*r*_. At this point, we are assuming that rewards are deterministic functions of the state where they are received.

The greedy maximization algorithm to obtain the optimal joint policy maximizing the state-value function of Eq (14) follows a message-passing scheme. Each agent selects its optimal action according to the local Q-table, then the agent forwards to the next agent in the RR schedule the list of positive probability state transitions after executing the selected action. Let the system start in a state *s*_0_, agent *δ*(1) selects $a_{1}=\underset{a}{\text{arg}\;\text{max}}\left\{Q^{\delta \left(1\right)}\left(s_{0},a\right)\right\}$ and sends a message $\left\langle s_{1}^{j},{\hat{P}}_{\delta \left(1\right)}\left(s_{0},a_{1},s_{1}^{j}\right);j=1,\ldots ,k_{1}\right\rangle$ to agent *δ*(2), where *k*_1_ is the number of states $s_{1}^{j}$ such that ${\hat{P}}_{\delta \left(1\right)}\left(s_{0},a_{1},s_{1}^{i}\right)>0$. Then, agent *δ*(2) selects its optimal action on the basis of the possible states:

$$\begin{array}{ccc} a_{2} & = & \underset{a}{\arg\;\max}\{\sum_{j=1\ldots k_{1}}{\hat{P}}_{\delta (1)}(s_{0},a_{1},s_{1}^{j})\\ & \cdot & ({\hat{R}}_{\delta (1)}(s_{1}^{i})+\gamma^{2}\cdot Q^{\delta (2)}(s_{1}^{j},a))\}, \end{array}$$

and sends a message $\left\langle s_{2}^{j},\hat{P}\left(s_{0},a_{1},,a_{2}s_{2}^{j}\right);j=1,\ldots ,k_{2}\right\rangle$ to the next agent *δ*(3), where

$$\begin{array}{c} \hat{P}(s_{0},a_{1},a_{2},s_{2}^{j})=\sum_{j=1\ldots k_{1}}{\hat{P}}_{\delta (1)}(s_{0},a_{1},s_{1}^{j})\cdot {\hat{P}}_{\delta (2)}(s_{1}^{j},a_{2},s_{2}^{j}), \end{array}$$

and *k*_2_ is the number of states $s_{2}^{i}$ such that $\hat{P}\left(s_{0},a_{1},a_{2},s_{2}^{i}\right)>0$.

Generalizing this process, agent *δ*(*i*) receives a message $\left\langle s_{i-1}^{j},\hat{P}\left(s_{0},a_{1}\ldots a_{i-1},s_{i-1}^{j}\right);j=1,\ldots ,k_{i-1}\right\rangle$, then selects its local optimal action

$$\begin{array}{ccc} a_{i} & = & \underset{a}{\arg\;\max}\{\sum_{j=1\ldots k_{i-1}}\hat{P}(s_{0},a_{1}\ldots a_{i-1},s_{i-1}^{j})\\ & \cdot & ({\hat{R}}_{\delta (i-1)}(s_{i-1}^{j})+\gamma^{i}\cdot Q^{\delta (i)}(s_{i-1}^{j},a)), \end{array}$$

and, finally, sends message $\left\langle s_{i}^{j},\hat{P}\left(s_{0},a_{1}\ldots a_{i},s_{i}^{j}\right);j=1,\ldots ,k_{i}\right\rangle$ to agent *δ*(*i* + 1). When the procedure reaches the last agent, all agents execute a joint-action **a** = [*a*_1_, *a*_2_, …, *a*_*N*_] with is the greedy solution to the following minimization problem:

$$\begin{array}{ccc} \pi_{D}(s) & = & \underset{a_{1},a_{2}\ldots a_{N}}{\arg\;\max}\{\sum_{j=1\ldots k_{N}}\hat{P}(s_{0},a_{1}\ldots a_{N},s_{N}^{j})\\ & \cdot & ({\hat{R}}_{\delta (N)}(s_{N}^{j})+\gamma^{N}\max_{a^{\prime }}Q^{\delta (N)}(s_{N}^{j},a^{\prime }))\}. \end{array}$$ (15)

For completeness, terminal states must be taken care of, because we are composing cycles of actions. If a terminal state is reached, there will not exist transition probability information from this state onwards. We can address this potential implementation issue by leaving unaltered the entries in the incoming messages for which no transition probability is available, so that the remaining agents will perform no action at all.

**Proposition 3** *The action selection policy defined by Eq (15) approximates the optimal greedy joint-policy given by Eq (8)*.

*Proof*. The transition and reward functions are approximated by $\text{P}\left(s,\text{a},s^{\prime }\right)≃\hat{P}\left(s,a_{\delta \left(1\right)},a_{\delta \left(2\right)}\ldots a_{\delta \left(N\right)},s^{\prime }\right)$ and $\text{R}\left(s,\text{a},s^{\prime }\right)≃\hat{R}\left(s,a_{\delta \left(1\right)},a_{\delta \left(2\right)}\ldots a_{\delta \left(N\right)},s^{\prime }\right),$ therefore, we can aggreagate local actions into a joint action, substituting both approximated terms in Eq (15) rewriting it as follows:

$$\begin{array}{ccc} \pi_{D}(s) & ≃\arg & \max_{a}\{P(s,a,s^{\prime })\cdot (R(s,a,s^{\prime })\\ & & +\;\gamma^{N}\max_{a^{\prime }}Q^{\delta (N)}(s^{\prime },a^{\prime }))\}. \end{array}$$

Furthermore, $\text{arg}\underset{a^{\prime }}{\text{max}}\;Q^{\delta \left(N\right)}\left(s^{\prime },a^{\prime }\right)=\text{arg}\underset{{\text{a}}^{\prime }}{\text{max}}\;{\text{Q}}^{\text{π}}\left(s^{\prime },a^{\prime }\right)$, we conclude that the joint policy approximates the optimal joint policy learned by a centralized learner given by Eq (8): **π**_*D*_(*s*) ≃ π*(*s*).

This approximation has been tested experimentally in environments with delayed rewards, with positive results. Unlike the technique presented in [25], where the message network could have different topologies, our message passing process only needs one cycle to finish. If our assumptions are completely fulfilled, the system is able to learn an optimal policy for < *T*∪*G*,**A** ^*e*^,**P**,**R** >.

### D-RR-QL with Modular State-Action Vetoes

An additional advantage of D-RR-QL is that it allows us to use MSAV in a multi-agent environments. Since we assume that a joint-action is approximately the same as the equivalent sequence of local actions, agents can veto actions on their local action space. Agents take actions one by one during the cycle of execution and thus have no interference from the rest of agents. Of course, this requires taking *N* time-steps to perform *N* local actions instead of a single joint-action, and the execution time will grow linearly to the number of agents. Nevertheless, this is neglectable in the case of overconstrained environments, where learning in an effective manner *A* ^*e*^(*s*) is critical.

The speed boost introduced by the use of MSAV in such multi-agent environments is even bigger than in the single-agent case (Section *Modular State-Action Vetoes*). Let the state be defined $S=S_{X}\times S_{i}^{U}$, being $S_{i}^{U}$ the state subspace used by the *i*-th *Veto-Module*, and let the joint-action space be defined **A** = *A*_*j*_ × *A* ^−^, where *A*_*j*_ is *j*-th agent’s local action space, and *A* ^−^ = *A*_1_ × *A*_*j*−1_ × *A*_*j* + 1_ × … × *A*_*N*_. Whenever an agent vetoes a state-action pair in its own state-action subspace, it is effectively vetoing ∣*S*_*X*_ × *A* ^−^ ∣ state-action pairs in the joint state-action space. The multi-agent Safe-MSAV architecture can be expected to avoid UTS much faster than any other exploration strategy operating on the complete state space *S*.

## Experiments

Linked Multi-Component Robotic Systems (L-MCRS) are over-constrained systems which can benefit from the use of D-RR-QL with MSAV. The hose transportation task is a paradigmatic application of L-MCRS: A set of *N* = 4 robots is attached to a hose at fixed points and the hose is modeled as a segment line lying between robots (Fig 3). This simplification of the original environment in which GEDS are used to model the hose is used as a training arena where agents learn the basic skills, and then they better tune the policies learnt in the GEDS environment. The goal is to transport the tip of the hose to a predefined goal position (the reward received is 10), while the other extreme is attached to a fixed position, a source, arbitrarily set at the center of the working space. The simulated world consists of 19 × 19 cells and each robot is controlled by an agent which can execute five actions: *A*_*i*_ = {*up*_*i*_, *down*_*i*_, *left*_*i*_, *right*_*i*_, *none*_*i*_}. The four constraints considered are the ones enumerated in Section *Problem Statement*, and whenever one of these termination conditions is met, the system generates a negative reward (−1) and the environment is reset to the initial setting (Fig 3). The system state is defined by the following state variables:

- The relative positions of the *N* robots. Relative positions are calculated with respect to the previous robot in the formation (source position (0,0) for the first robot).
- Self-position in absolute coordinates. Each robot perceives its own absolute position.
- Four flag variables indicating whether there is an obstacle (hose, robot) in each of the four possible directions a robot can move.

Benchmark algorithms (D-QL, Team-QL and Coordinated-RL) only use robot relative positions because the remaining state variables are a linear function of them and these algorithms cannot benefit from the additional information provided by the extended set of variables. For instance, it is of no use to include the obstacle flag variables because they are a function of the relative positions of the robots and thus, can only take one value for any given set of relative positions. In order to best learn how to avoid breaking a physical constraint corresponding to an UTS, we used three Veto-modules within each agent’s MSAV policiy, each of which in charge of a specific physical constraint:

- Overstretching the hose. This module receives a negative reward every time a hose segment is overstretched, and its local state space encompasses the relative positions of the robots preceding and following the robot which executed the action that led to over-stretching its hose segment.
- Collisions. Every time a robot takes an action that makes it collide with any other, this module receives a negative reward. The local state space is composed by the obstacle flag variables.
- Simulation bounds. This last module uses only its robot’s absolute position and receives a negative reward every time a robot gets out of the simulation grid.

Each of the four generated negative rewards was fed to a specific module learning the veto of specific undesired state-actions using the value of the *relevant state variables* [8].

Because the amount of episodes and the degree of exploration is key to this particular kind of tasks, experiments were run with different number of episodes *n*_*e*_. D-RR-QL with MSAV was run using the Safe Soft-Max policy Eq (6), while the rest of algorithms used regular Soft-Max exploration Eq (4). We also tested *ϵ* − *greedy* policies but the experiments yielded slightly worse results and thus, will not be reported here. Initially, the temperature parameter was set *τ* = 10 and decreased at the end of each episode with △*τ* = −10/*n*_*e*_. Starting from episode number 500, the learnt policy was evaluated using greedy action selection each 500 episodes. This is the reason why data plots start from episode 500 instead of episode 0. Each experiment was run 3 times and the results were averaged. The performance was measured using two different criteria: (a) the accumulated discounted rewards, which is the typical performance measurement in the RL field, and (b) the number of cells reached with the tip of the hose, which allows us to intuitively assess how effective the exploration of the state-space is.

We implemented ourselves these experiments in C/C++. The source code used in our experiments and can be accesed publicly at: http://www.ehu.es/ccwintco/index.php?title=D-RR-QL.

### Experiment 1

**Fig 4:**
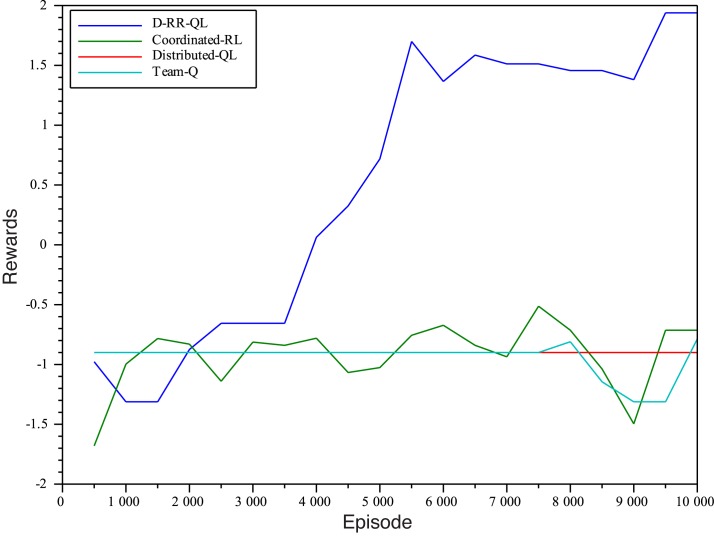
Rewards obtained by the learnt greedy policies with *n*_*e*_ = 10^4^.

**Fig 5:**
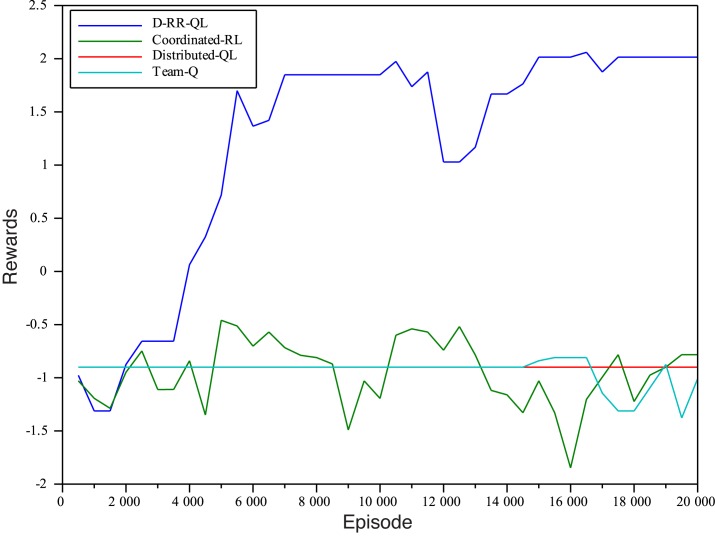
Rewards obtained by the learnt greedy policies with *n*_*e*_ = 2 ⋅ 10^4^.

In this first experiment, we tested two different values of *n*_*e*_ = {10^4^,2 ⋅ 10^4^}. Figs 4 and 5 show the accumulated rewards obtained by agents in the two different settings. The learning gain by using D-RR-QL with MSAV is huge. In both cases, it is the only algorithm able to obtain a positive accumulated discounted reward in the evaluation episodes. It only requires about 4,000 episodes for our method to learn a greedy policy that can reach the goal position. The rest of algorithms are not only incapable of avoiding UTS, but also of *even reaching the goal cell a single time*.

**Fig 6:**
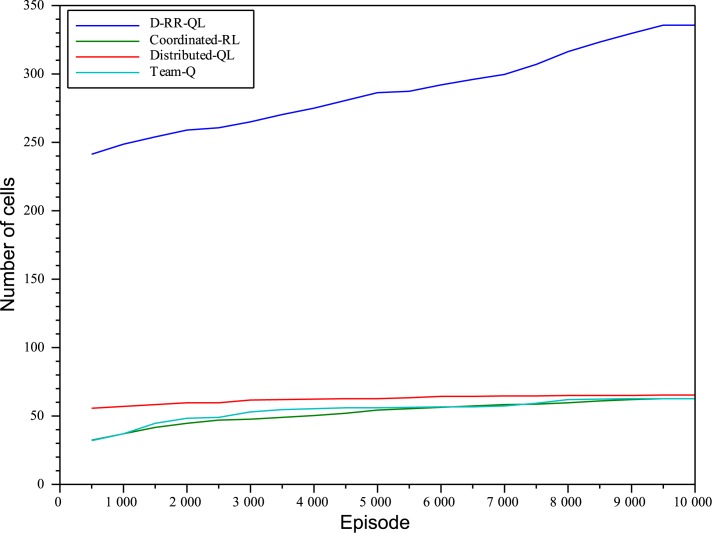
Number of cells reached with the tip of the hose with *n*_*e*_ = 10^4^, separate plots when applying D-RR-QL, Distributed-QL, Coordinated-RL, and Team-QL algorithms.

**Fig 7:**
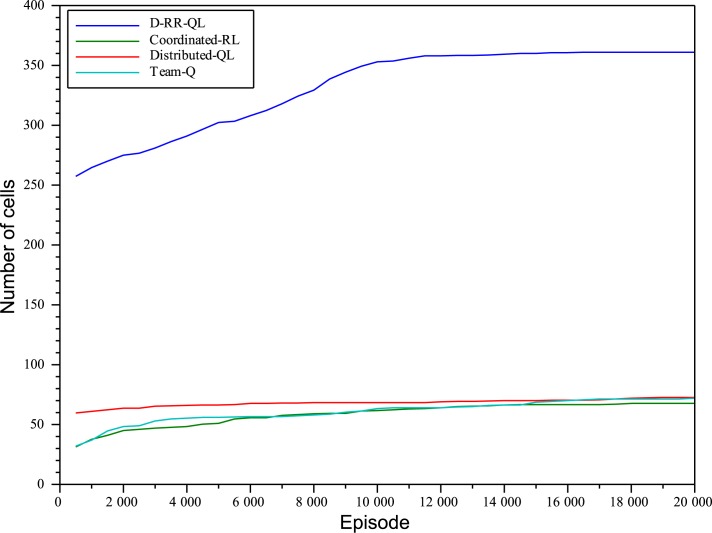
Number of cells reached with the tip of the hose with *n*_*e*_ = 2 ⋅ 10^4^, separate plots when applying D-RR-QL, Distributed-QL, Coordinated-RL, and Team-QL algorithms.

Figs 6 and 7 on the other hand show the number of different states reached with the tip of the hose. The plot clearly shows that the exploration of the state-space is much more efficient when using MSAV than by means of standard Soft-Max policies. By the time D-RR-QL has been able to learn a greedy policy able to reach the goal, the system has already reached about 275 different cells with the tip of the hose. On the other hand, none of the other algorithms is able to reach more than 70 different cells throughout the complete experiment.

### Experiment 2

In order to get a clearer idea of how far the benchmark algorithms are from the goal of learning a greedy policy that reaches the goal position, we ran a second set of experiments where agents were allowed much more time for exploration of the environment (*n*_*e*_ = 5 ⋅ 10^5^ and *n*_*e*_ = 10^6^). We only report the number of reached cells with the tip of the hose, because the accumulated discounted rewards are similar to those obtained in the previous experiment, and offer no farther information. Both Soft-Max and *ϵ* − *greedy* policies were tested and, in this case, slightly better results were obtained by using *ϵ* − *greedy*. Initially, *ϵ* = 1 and every episode it was decreased with △*ϵ* = −1/*n*_*e*_. This is paradoxical considering the better results obtained by Soft-Max policies in the previous experiment. Our educated guess is that this is due to using the same decay schedule for the two exploration parameters *ϵ* and *τ*. While they have a similar function, *ϵ* is the probability of exploring each time-step and *τ* shapes the probability distribution of the action selection algorithm. Moreover, as the Soft-Max algorithm depends on the magnitude on the actual estimations of the value function, the efficiency of the exploration algorithm needs not improve linearly to the number of episodes allowed to the agents.

**Fig 8:**
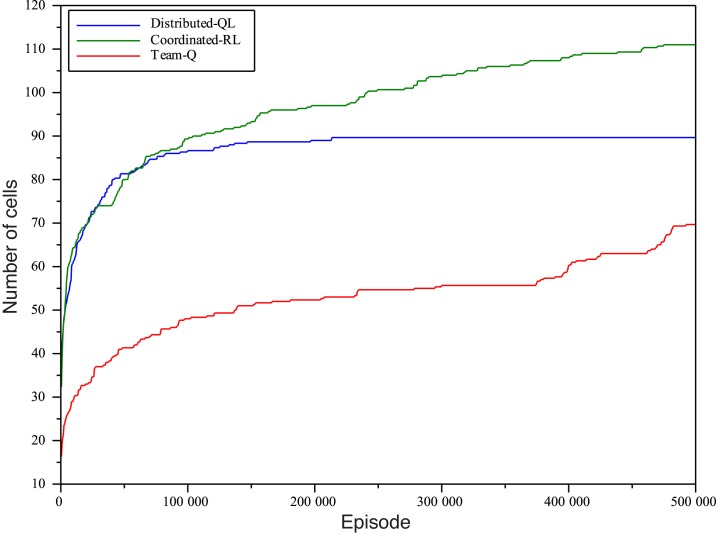
Number of cells reached with the tip of the hose with *n*_*e*_ = 5 ⋅ 10^5^, separate plots when applying Distributed-QL, Coordinated-RL, and Team-QL algorithms.

**Fig 9:**
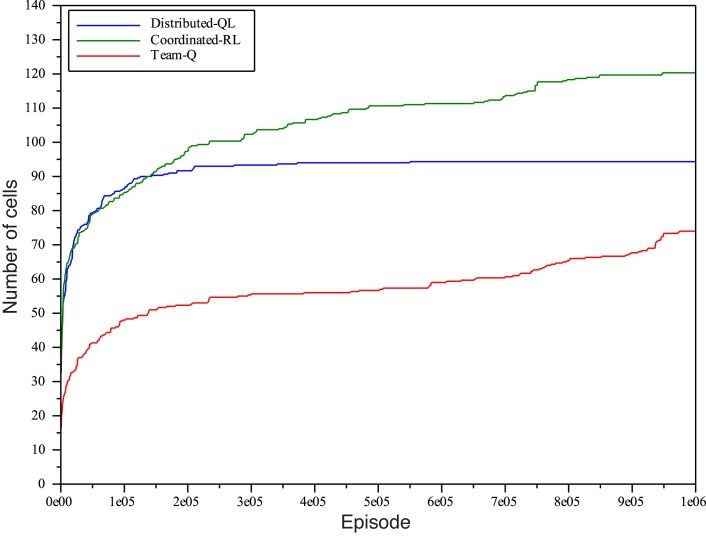
Number of cells reached with the tip of the hose with *n*_*e*_ = 10^6^, separate plots when applying Distributed-QL, Coordinated-RL, and Team-QL algorithms.

Figs 8 and 9 plot the number of cells and, although the maximum amount of episodes is 2 orders or magnitude greater than in the previous experiments, the system is only able to reach about 120 cells with the mobile tip of the hose in the case of Coordinated-RL, which outperforms the other two algorithms. Next comes Distributed-QL with 92 cells visited, and finally, Team-QL is only able to reach 74 cells. It is very important to notice that, once again, *neither of these algorithms was able to reach the goal cell even once* during the experiments. For the sake of comparison with the D-RR-QL and MSAV approach, let us recall that the algorithm was first able to reach the goal greedily selecting the best action when 275 cells had been visited with the tip of the hose. This is a clear indication of how inefficient the exploration of over-constrained environments by standard action selection policies is: if we take the number of cells visited by D-RR-QL before it can learn to reach the goal cell as a reference of how much exploration is needed before a goal state has been discovered, all the competing algorithms are very far from this number even if allowed to explore two orders of magnitude more.

## Conclusions

This paper formalizes and gives convergence proof of a distributed Q-Learning algorithm for MARL problems amenable to embed Modular State-Action Veto (MSAV) policies which improve performance in over-constrained environments. First, we have presented agent interaction model as a Cooperative Round Robin Stochastic Game (C-RR-SG), in which agents select and take actions following some predefined order. A communication-free distributed implementation called Distributed Round Robin Q-learning (D-RR-QL) has been proposed, providing a proof of its convergence to the optimal policy on a C-RR-SG. We have also presented a message-based procedure to obtain the optimal policy for the original cooperative stochastic game (C-SG) from the local policies learned by D-RR-QL. We give a consensus-based implementation for the decentralized determination of RR turns that may allow fully distributed implementation of the D-RR-QL.

Computational experiments show that D-RR-QL using MSAV policies can provide a valid joint-action policy approximating the optimal policy faster than D-QL, Team-QL, and Coordinated-RL in over-constrained systems. Furthermore, the D-RR-QL convergence is not limited to deterministic MDPs, it can also cope with stochastic environments. Communication requirements to learn local Q-values (*O*(1) messages each step if communications are used, 0 messages otherwise) and to obtain the global policy deciding a joint-action (each action requires *O*(*N*) messages) are minimal when compared to coordinated multi-agent approaches such as Coordinated-QL. As a line of future work, we need to study the equivalence between C-SG and C-RR-SG to determine the degree of approximation that can be obtained when there is no C-RR-SG equivalent to a C-SG. We also plan to investigate more general ways to learn how to avoid reaching undesirable terminal states. On the other hand, the degree of abstraction used in our experiments limits the applicability to real robot control problems. This approach can be directly applied in real environment as long as low-level controllers and sensors provide this kind of abstract actions and states, but the coarseness of the state-action space representation limits the optimality of the learned policies. Therefore, we plan to use continuous state-action spaces in our future research.
